# Bridging the gap between patient and physician perspectives on management of generalized myasthenia gravis: a Delphi consensus study

**DOI:** 10.1186/s13023-026-04312-7

**Published:** 2026-03-20

**Authors:** Andrew Chan, Monika Kaempf, Wolfgang N. Löscher, John Vissing, Johan Voerman, Eva Frostell-Pyhäjärvi, Sari Atula

**Affiliations:** 1 Department of Neurology, Inselspital, Bern University Hospital, University of Bern, Bern, Switzerland; 2 ASRIMM, Association for Neuromuscular Diseases covering Swiss French Cantons, Yverdon-les-Bains, Switzerland; 3 Department of Neurology, Medical University Innsbruck, Innsbruck, Austria; 4 Copenhagen Neuromuscular Center, Rigshospitalet, University of Copenhagen, Copenhagen, Denmark; 5 Diagnosis Working Group, Spierziekten Netherlands, Delft, Netherlands; 6 Finnish MG Association, Ex-European Myasthenia Gravis Association, Vantaa, Finland; 7 HUS Neurocenter, University of Helsinki, Helsinki, Finland

**Keywords:** Generalized myasthenia gravis, Delphi consensus, Quality of life, Europe

## Abstract

**Background:**

There is currently limited data to guide treatment selection, dosing, combination strategies and sequencing in the management of myasthenia gravis (MG). Additionally, MG symptoms are heterogenous between people and with time, requiring an individualized treatment approach. As such, successful patient-physician communication is key. This Delphi project was undertaken by MG specialists and patient representatives to explore their opinion on improving MG management via effective physician-patient communication and collaboration approach.

**Methods:**

This mixed-methods study was conducted in two phases. In Phase I, seven MG specialists and two patient representatives contributed to idea generation. Relevant insights informed the development of the Phase II Delphi survey. A panel of 16 MG specialists and seven patient representatives participated in a two-round Delphi survey. Consensus was defined as ≥ 70% agreement or disagreement on a 6-point Likert scale. Participants responded to the survey from what they considered an ideal scenario, regardless of their real-world experience.

**Results:**

Consensus was achieved on 89% of all statements. Key areas of alignment were – importance of incorporating patient preferences on quality of life (QoL), sustained symptom control, route of administration, and side effects profile. Both groups agreed that current clinical practices insufficiently integrate mental health considerations and patient engagement remains suboptimal. Two key areas of misalignment were – patient representative and physicians had different perspective on how well physicians understood patient preferences and both groups showed different interpretations of side effects (adverse reaction versus issues of tolerability).

**Conclusion:**

This Delphi consensus found that while physician and patient representatives shared similar perspectives these were not always reflected in current clinical practice due to differences in understanding of patient preferences. Prioritizing structured conversations on QoL, treatment expectations, and side effect were found important for physicians while improving access to information, openly communicating and actively participating in treatment decisions were key outcomes for patients. This study lays a valuable foundation for deepening conversations and alignment on key topics in MG management among the medical, patient and caregiving communities.

**Supplementary Information:**

The online version contains supplementary material available at 10.1186/s13023-026-04312-7.

## Background

Myasthenia Gravis (MG) is a rare and chronic autoimmune disease mediated by immunoglobulin autoantibodies (IgG). It is characterized by impairment of neuromuscular transmission at the postsynaptic membrane of the neuromuscular junction (NMJ), leading to fatigability of skeletal muscles and fluctuating muscle weakness, which worsen with exertion and improve with rest [1, 2]. Typically, symptoms manifest first in ocular muscles, with ptosis (drooping eyes) and diplopia (double vision) as commonly occurring symptoms. In over 80%, however, symptoms generalize (generalized MG, gMG) to other muscle groups such as bulbar, axial, limb and respiratory causing limb weakness, difficulties in walking and shortness of breath, among others [3, 4].

There is symptomatic treatment, but no cure for MG. Additionally, the heterogeneity of MG symptoms, characterized by variability between patients and over time, warrants an individualized approach to disease management. With appropriate symptomatic management, improved immunotherapy and care, most people living with MG can have improved quality of life (QoL) [5]. Current treatment options include acetylcholinesterase inhibitors (AChEI), glucocorticoids (GC), intravenous immunoglobulin (IVIg), plasma exchange (PLEX), thymectomy, and immunosuppressive agents, such as rituximab. Among the 10% of people who do not respond to these treatments, the recently introduced innovative, novel therapies such as complement inhibitors (C5i), and neonatal Fc receptor inhibitors (FcRni) could offer promising benefit/risk outcomes [6–12].

Timely access to appropriate treatment is critical to MG management, as worsening symptoms affect QoL and can lead to exacerbation or crisis, posing significant patient and healthcare resource utilization burden [13, 14]. Despite the increasing number of immunomodulatory treatments, currently there is only limited data on treatment selection, combination and sequencing, underlining the need for individualized treatment approaches. Effective management, thus, depends on successful patient-physician communication. However, evidence suggests that patients may be left dissatisfied with healthcare provider (HCP) interactions, reporting inadequate understanding of patient preferences, the extent of impact on their QoL and overall functioning as well as “treatment inertia” [15–17]. Conversely, HCPs report challenges in managing persistent symptoms such as profound lack of energy, which do not respond well to treatments and may be attributable to mechanisms other than neuromuscular impairment. Correspondingly, they may have a cautionary approach towards treatment modification due to concerns for adverse effects or uncertainty [13, 18]. This misalignment can result in maintenance of treatment regimens that compromise patient preferences [19, 20].

This Delphi consensus project was a joint undertaking of MG specialists and MG patient advocacy group (PAG)/patient representatives to explore how effectively physicians and patients communicate and align in decision making for gMG management. The current study involved HCPs and patient representatives in Central and Northern Europe (CENE) to co-create real-world evidence, particularly to investigate patient needs and perspectives, and understand how well these preferences are incorporated into treatment decision making. Given the involvement of MG-specialists and PAG representatives who closely support people living with gMG, including a few PAG representatives with personal experience of living with gMG, this study contrasts the real-world experiences of both groups with their opinion of an “ideal” approach to physician-patient communication and collaboration. In doing so, the panel aims to pave the way for improved patient involvement and effective decision making in gMG management.

## Methods

### Overview

This Delphi consensus project comprised two-phases wherein Phase I focused on idea generation, while Phase II was a two-round Delphi survey. Phase I was conducted through an international advisory board (AdBoard), with a panel of seven specialized HCPs and two PAG representatives. The panel convened for an online discussion in June 2024. The meeting proceedings informed Phase II, the Delphi survey. The Delphi survey was hosted on a specialized online platform and conducted in two consecutive rounds over a three-month period at the end of 2024. An expanded panel of experts comprising 16 HCPs and seven PAG representatives participated in both rounds of the Delphi survey. Six HCPs and two PAG representatives had previously participated in the AdBoard. The study used mixed methods, collecting qualitative data (open-ended questions and responses summarized qualitatively) and quantitative data (rating scales summarized quantitatively).

**Fig. 1:**
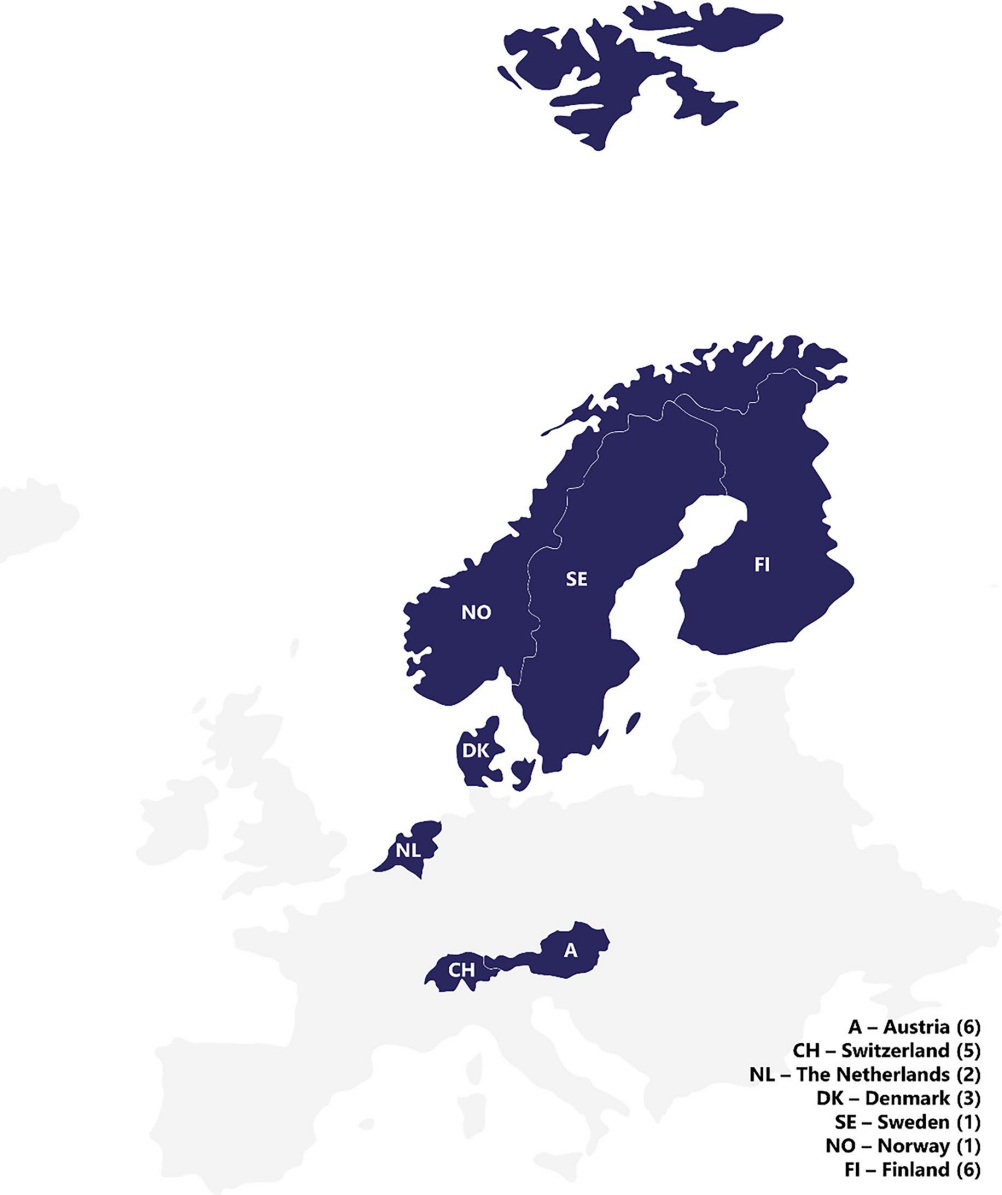
Overview of the participants in both phases of the study. The number of participants is indicated in country name (N), where N indicates the total of healthcare providers and patient advocacy group representatives

**Fig. 2:**
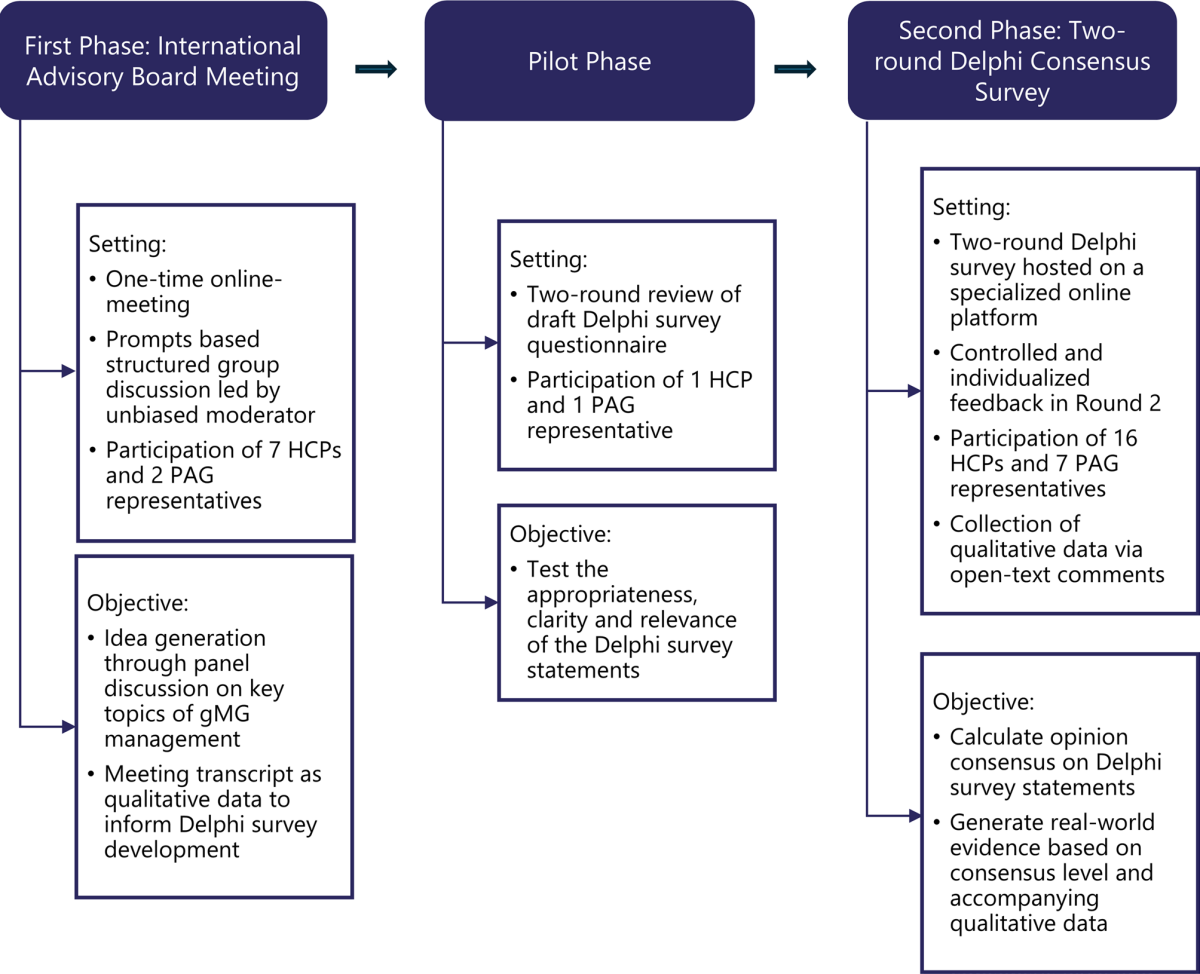
Research methodology overview of this two-phase delphi consensus project. Phase I generated ideas via an international advisory board meeting of a panel of experts including healthcare professionals and patient representatives. Phase II was a two-phase delphi survey with an expanded panel of experts

The primary criteria for participation were the regional focus on CENE countries (Fig. 1) with English as a language requirement. All HCPs were medical doctors specialized in neurological or neuro-muscular disorders including gMG management in their respective countries, with anywhere from 10 to over 30 years of clinical experience in treating patients with gMG. HCPs were involved in the treatment of patients in hospitals, specialized center or private practice settings. The PAG representatives had decades of collaboration with their patient organization, and relevant experience in advocating and supporting people living with gMG. The following Fig. 2 reports the key aspects of the research methodology.

### Survey development

The AdBoard, led by a trained moderator, conducted an open discussion between the HCP and PAG representative groups on topics related to management of gMG. HCP and PAG-specific prompts on issues such as patient needs, patient-physician communication, treatment goals and approach were utilized to guide the discussion. Qualitative data from the meeting transcript was thematically assessed to identify new considerations, current gaps in gMG management as well to confirm findings that were known via prior research. These findings, particularly the gaps and new findings, were used to develop the Delphi survey. The draft survey was pilot tested with one HCP and one PAG representative each to ensure the clarity, appropriateness and validity of the questions.

### Survey execution

The specialized online platform hosting the survey enabled anonymous participation. The survey questionnaire utilized a 6-point Likert scale (strongly disagree, disagree, somewhat disagree, somewhat agree, agree, strongly agree) with an additional option for free-text comments. Round 1 was launched in mid-October 2024, and Round 2 was launched in early December 2024. Both rounds were completed in a 4-week response period. A positive medium consensus was defined when ≥ 70% of participants agreed or strongly agreed to a statement. With ≥ 80% agreement level, a positive high consensus was obtained. Conversely, when ≥ 70% and ≥ 80% of participants disagreed or strongly disagreed with a statement, negative medium and negative high consensus, respectively, was obtained. An alignment of opinions between HCPs and PAG representatives was determined if at least 70% of each group either agreed or disagreed with a statement. Alternatively, if one group reached a positive consensus and the other a negative consensus or, if one group achieved consensus and the other did not, it was concluded that the groups were of differing opinion. Statements that had obtained at least 70% consensus in Round 1 were not included in Round 2 of the survey. More importantly, Round 2 featured individualized questionnaires where each participant could view a statistical report of their individual and group responses from Round 1, including the mean, mode, median, and frequency distribution along the 6 Likert-scales. This controlled feedback allowed participants to compare their individual response to the group response.

All the participants were requested to record their responses considering the ideal scenario, even when this did not reflect their current real-life experiences. Table 1 outlines the role and responsibilities assumed by participants through the conduct of this research.

**Table 1:** Delphi Consensus project participants and their roles

Study Phase	Participant (*N*)	Role
International Advisory Board	Healthcare providers (HCPs) (7)	The participating HCPs supported idea generation on key aspects of gMG management by responding to discussion prompts. They participated in a structured group discussion and gave clinical and real-world context to inform design of Delphi survey.
International Advisory Board	Patient Advocacy Group (PAG)/patient representatives (2)	The participating PAG representative supported idea generation on key aspects of gMG management by responding to discussion prompts. They participated in a structured group discussion and gave real-world context from the patient perspective to inform the design of Delphi survey.
Delphi Survey Pilot	HCP (1)	This HCP reviewed, edited and finalized the Delphi Survey draft ensuring the medical correctness and clarity of the questions.
Delphi Survey Pilot	PAG representative (1)	This PAG representative reviewed, edited and finalized the Delphi Survey draft ensuring the appropriateness, comprehensiveness and clarity of questions from a patient perspective.
Delphi Consensus Survey Rounds	HCPs (16)	The participating HCPs responded to the Delphi surveys and provided qualitative data via comments to support their opinion. As leading national experts in gMG management, their participation produced real-world evidence to determine consensus outcomes.
Delphi Consensus Survey Rounds	PAG representatives (7)	The PAG representatives responded to the Delphi surveys and provided qualitative data via comments to support their opinion. As leading patient advocates in their national context, their input produced the evidence which informed the consensus outcomes.
Independent Support	Clinical research organization & Sponsor (4)	Cytel, an independent clinical research organization based in Germany supported the Delphi survey development and conduct. Alexion, AstraZeneca Rare Disease supported as the study sponsor, providing guidance to identify relevant HCPs.

Abbreviations: gMG, generalized myasthenia gravis; HCP, healthcare provider; PAG, patient advocacy group

## Results

### Consensus statements

**Fig. 3:**
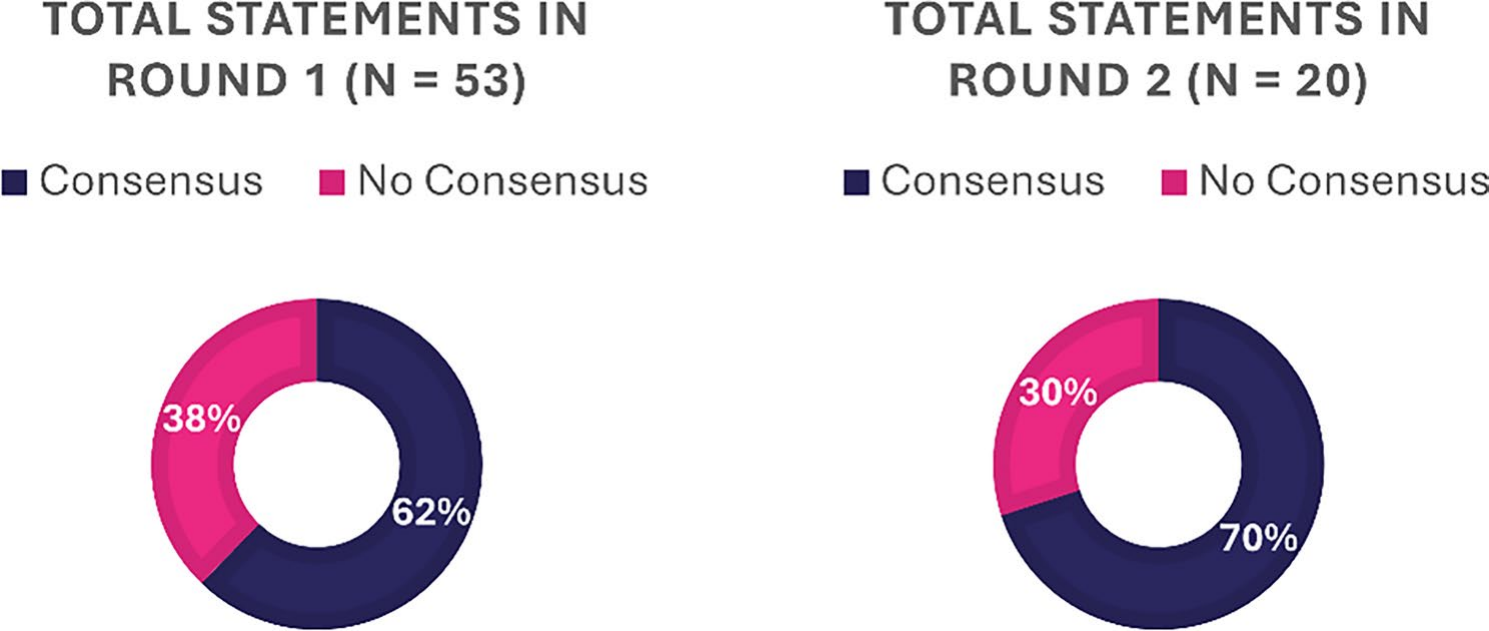
Overview of Delphi consensus results. The Delphi survey questionnaire contained 53 statements from 21 questions. Of these, 62% of statements that obtained consensus in Round 1 were not included in Round 2. Round 2 survey contained 20 statements of which 70% obtained consensus

The Delphi Survey questionnaire contained 21 questions. As one question could contain multiple parts, the 21 questions produced 53 individual statements. Both rounds of the survey were concluded with a 100% completion rate. At the end of Round 1, 62% of statements (33 of 53) achieved consensus. The remaining 20 items that did not achieve consensus in Round 1 were carried forward to Round 2. In Round 2, 70% of statements (14 of 20) of the statements achieved consensus. Thus, at the end of both rounds, 89% of the statements (47 of 53) had achieved consensus. Notably, none of the statements achieved a negative consensus, meaning no statement had 70% or more participants disagree with it. The results overview is presented in Fig. 3, with full results available in supplementary material.

### Patient needs & perspectives

To investigate patient needs and perspectives, six survey questions addressing these topics were analyzed. These six questions contributed to 20 statements, of which 90% (18 of 20) achieved consensus. Of the 18 statements that achieved consensus, HCPs and PAG representatives were of the same opinion on 17 statements (meaning at least 70% HCPs and 70% PAG representatives agreed with the statement). Table 2 presents the results of these Delphi statements.

**Table 2:** Delphi survey questions investigating patient needs and preferences

*N*	Delphi Survey Question/Statement	Overall Consensus^†^ (%) (*N* = 23)	Consensus (%) HCPs (*N* = 16)	Consensus (%) PAG representatives (*N* = 7)
1	When selecting gMG treatments, these patient preferences are considered: a) Sustained symptom control b) Safety c) Route of administration d) Dosing frequency e) Necessity of treatment side effects monitoring (e.g., regular blood tests)	100% 96% 91% 83% 74%	100% 94% 94% 88% 75%	100% 100% 86% 71% 75%
2	Mental health-comorbidities such as depression or anxiety disorder are equally important as physical health-comorbidities such as diabetes when making treatment decisions.	83%	88%	71%
3	In gMG care, the potential for quality-of-life improvement is a compelling reason to initiate or modify treatments, even for patients with mild or stable clinical profiles.	83%	81%	86%
4	There is utility for novel therapies if gMG symptoms are well controlled and a) There are potential short or long-term side effects of the current treatment. b) There is some potential to improve quality of life c) There are minor long-term side effects of the current treatment d) There is limited potential to improve quality of life e) There are minor short-term side effects of the current treatment	83% 74% 70% No consensus No consensus	88% 75% 75% 25% 19%	71% 71% 57% 29% 43%
5	When determining the potential for gMG treatment initiation or modification to improve quality of life, following aspects are important components a) Physical capabilities and daily functioning b) Professional functioning and career impact c) Social well-being and relationships d) Mental health and emotional state e) Overall life satisfaction	100% 91% 91% 87% 83%	100% 94% 88% 81% 88%	100% 86% 100% 100% 71%
6	The following strongly indicates the timing for reevaluating and modifying the current treatment regimen or dosage a) Patient dissatisfaction with treatment side effects b) Unmet treatment goals within set timelines c) Patient dissatisfaction with their quality-of-life	87% 78% 78%	94% 81% 81%	71% 71% 71%

The overall consensus level was derived by calculating the percentage of participants (Total *N* = 23) who agreed or strongly agreed with a statement. Similarly, group consensus for healthcare providers and patient advocacy group representatives were calculated using total *N* = 16 and *N* = 7, respectively†The statements are ordered by % consensus to improve readability. The panelists may have responded to a different order of statements in the original survey questionnaire as indicated in the Supplementary Material

With a high positive consensus, both HCPs and PAG representatives supported patient preference for sustained symptom control and safety profile when selecting suitable treatments. In their comments, HCPs reported high regard for patient input in prioritizing treatment considerations, underlining this as an “ideal” to ensure treatment adherence. HCPs also supported route of administration as an important factor due to its adherence benefits, noting that easy-to-take or self-injected treatments could improve disease management, particularly for people with professional and familial responsibilities. Multiple HCP comments also acknowledged patient willingness to compromise preferences when the benefit/risk outcomes appear favorable. This was echoed in PAG representatives’ comments stating that patients may view treatment choices as “*beyond patient control*”, with readiness to compromise preference in return for symptomatic control.

Statement 2, which focused on whether mental health comorbidities were considered equally important as physical comorbidities in treatment decisions, achieved a high consensus from HCPs but only a medium consensus from PAG representatives. In part, this was explained by the PAG representative comments indicating fewer mental health-related communication with physicians. Nonetheless, other comments confirmed that patient experiences of bad mental health days often correlated with a worse experience of gMG symptoms. HCPs commented that holistic patient care should prioritize all aspects of health, noting role of mental health comorbidities in treatment adherence. HCPs raised three key considerations– severity of certain mental health comorbidities, the interaction of gMG and comorbidity medication and the effect of gMG medication, such as steroid use, on mental health. Both HCPs and PAG representatives recognized that currently mental health was not considered as highly as it should.

Statement 3 stated that the potential for QoL improvement was a compelling reason for treatment modification, even for patients with stable clinical profiles. Comments revealed critical differences in expert opinion in ideal scenario and real-world experience. PAG representatives commented that HCPs hesitate to switch treatment in case of mild symptoms, noting the role of cost considerations in HCP decisions. HCPs commented that on average clinical improvement correlated with QoL improvement, but in contrary event, thorough assessment of underlying cause of dissatisfactory QoL would precede a treatment switch. Nonetheless, several HCPs, using patient examples, commented that clinical improvement may still leave space for QoL improvement.

Statement 4 focused on clinically stable patient profiles who could benefit from novel treatments. On all statements that achieved consensus (sub-part statement 4b, 4d, 4e), a higher consensus was obtained among HCPs than PAG representatives. These results were explained by comments indicating a lower novel therapy-related knowledge among PAG members. Further, HCPs achieved consensus on novel therapy utility when there were minor long-term side effects of the current treatment, while PAG representatives did not. PAG representative comments iterated real-world experience of patients frequently compromising on minor side effects in exchange for symptom control. However, PAG representatives commented that several potential short or long-term effects, such as cancer or bad vision could be particularly worrying for patients.

Comments received from statements 5 and 6 further reiterated the importance of holistic treatment approach with high priority for patient preference in decision-making.

### Patient preference & treatment decision-making

To investigate if patient preferences are incorporated into treatment decision making, five questions addressing this topic were analyzed. In all, these contributed to 11 statements, of which 91% (10 of 11) achieved consensus. Of these 10 statements that achieved consensus, HCPs and PAG representatives were of the same opinion on six statements (meaning at least 70% HCPs and 70% PAG representatives agreed to the statement). Table 3 presents the results of these Delphi statements.

**Table 3:** Delphi survey questions directed at investigating if patient needs are incorporated into treatment decision making

*N*	Delphi Survey Question/Statement	Overall Consensus^†^ (%) (*N* = 23)	Consensus among HCPs (%) (*N* = 16)	Consensus among PAG representatives (%) (*N* = 7)
1	At the time of treatment initiation, patients and physicians openly discuss and align on key aspects of the treatment journey including, a) waiting time to observe treatment effects b) overall treatment goal for the patient c) treatment goal for the currently prescribed therapy d) potential side effects of currently prescribed therapy e) time to switch to a new treatment when set goals are not achieved f) definition of treatment effectiveness used in assessing treatment outcomes	96% 91% 91% 91% 83% 74%	94% 94% 88% 100% 70% 80%	100% 86% 100% 71% 50% 50%
2	Transparent discussions between patients and physicians considerably improve a) patient education about current treatment options and practices b) physician understanding of patient preferences and treatment priorities	87% 83%	88% 94%	86% 57%
3	There is a need to enhance the level of open and transparent communication between physicians and patients.	78%	81%	71%
4	Patient dissatisfaction alone is sufficient to consider treatment switch.	No consensus	53%	57%
5	Patient dissatisfaction with treatment side effects is as important as dissatisfaction with efficacy in guiding treatment modifications.	74%	88%	43%

The overall consensus level was derived by calculating the percentage of participants (Total *N* = 23) who agreed or strongly agreed with a statement. Similarly, group consensus for healthcare providers and patient advocacy group representatives were calculated using total *N* = 16 and *N* = 7, respectively†The statements are ordered by % consensus to improve readability. The panelists may have responded to a different order of statements in the original survey questionnaire as indicated in the Supplementary Material

Both HCPs and PAG representatives, with high consensus, supported the discussion of treatment goals and waiting time at treatment initiation in an ideal scenario. HCP comments highlighted the role of such discussions in setting clear expectations and avoiding complications in follow-up, while recognizing the present shortcomings in integrating this into clinical practice. Among the HCPs, strongest support was achieved on openly discussing treatment side effects. Further, HCPs acknowledged that even among practitioners there were few standardized ways to capture or align on treatment effectiveness definition, making it an even more challenging topic to address with patients. PAG representative noted that at treatment initiation patients may feel overwhelmed and thus, prioritize treatment, even if it came at a “*price*”. Importantly, their comments highlighted that patients perceive open discussion on all side effects to occur only when patients themselves initiated it.

Statement two focused on the relevance of transparent communication between HCPs and patients. Comments indicated that both groups agreed that transparent communication was necessary for patients to understand their disease and its management. More importantly, comments noted that improved knowledge was necessary for patients to clearly direct their preferences to doctors. HCPs were also of the opinion that ideally transparent communication would help them better understand patient preferences. However, patient representatives did not reach consensus on this statement. Other comments noted that in practice, communication was hindered by a lack of ample consulting time, differences in practices between doctors and transition between multiple specialists over the course of patient care. Additionally, it was noted that patient communication at specialized centers tended to be more informative than those held at individual practices, leading to inconsistent patient experiences in different settings.

Neither HCPs nor PAG representatives obtained consensus on the sufficiency of patient dissatisfaction alone to consider treatment switch. Both groups commented that treatment switch should be considered only when substantial gains are expected. HCP comments highlighted cost and regulatory approval constraints.

With a high consensus, HCPs found that patient dissatisfaction with treatment side effects was as important a consideration as their dissatisfaction with efficacy. However, from a PAG perspective no consensus was obtained. Their comments stated that patients may be prepared to tolerate minor side effects if the treatment improved the symptoms of MG.

## Discussion

Over recent years, the treatment landscape for gMG has significantly evolved. People living with gMG can now benefit from various available treatments for symptomatic and disease management, along with the innovative, novel therapies targeting specific pathophysiological mechanisms. Nonetheless, given the fluctuating nature of the disease, the standard treatment involves ongoing adjustments to identify an individualized optimal treatment-dose combination. Against this background, this panel of experts identified the critical role of effective physician-patient communication in successfully managing gMG. This study focused on identifying patient needs and investigating their integration in healthcare decision making. In particular, by drawing on opinion consensus among HCPs and comparing it to those reported among PAG/patient representatives, this Delphi consensus project contrasted how well the opinions of these stakeholders align in both real-life and ideal scenarios.

A positive finding of this study was the strong alignment between HCPs and PAG representatives regarding key patient preferences in an ideal setting. Both groups consistently reported patient preference for sustained symptom control, route of administration and safety outcomes. QoL emerged as a key patient priority, something that HCPs recognized and promoted. For example, 75% of HCPs and 71% of PAG representatives agreed that even with well-controlled symptoms, there is utility in switching to novel therapy, if the QoL is perceivably low. Similarly, 81% of HCPs and 71% of PAG representatives agreed that patient dissatisfaction with QoL is a strong reason for treatment revaluation. Despite this alignment, real-world evidence presents a contrasting picture. Patient surveys in several European countries find that people living with MG, despite receiving recommended therapy, report low QoL including hindrance to employment and mental-wellbeing leading to additional emotional and financial burden [14, 21]. These findings underline a disconnect between an ideal treatment approach and everyday clinical outcomes, calling for better integration of patient priorities and QoL in treatment decision, as well as the need for newer therapies that could better address current patient unmet needs.

Relatedly, a critical finding of this research was the non-consensus among PAG representatives regarding physician understanding of patient preference and treatment priority. At least 94% of HCPs were of the opinion that following transparent communication, they have an improved understanding of patient preferences, whereas only 57% PAG representatives supported this statement. This indicates that patients perceive severe gaps in how well their physicians understand their perspectives, which likely results in patient dissatisfaction with the integration of these preferences in treatment decision making. This finding echoes outcomes from a physician and patient survey, wherein 64% of HCPs reported being “very satisfied” with their patient’s treatment compared to only 39% of patients – a sharp difference of perspective [22]. Other engagements with patients have also found evidence of patients perceiving lack of understanding from their physicians due to suboptimal communication [15, 23]. Such persistent disparities undermine physician-patient trust and result in poorer long-term management.

PAG representative comments underlined further evidence of patient dissatisfaction, for example, in their report of insufficient consulting time, or systematic differences in communication at specialized centers versus individual practices, with patients expressing preference for specialized centers. Such reports indicate two issues – one concerning the quantity of time spent on consultations and another concerning the quality of meaningful and transparent dialogue between the parties. Notably, some of these challenges stem from systemic constraints of a national healthcare setting. For example, limited time per consultation, lack of treatment continuity by a single specialist or uneven access for patients to specialized centers are factors inherent to a national healthcare setting, and despite patient and doctor alignment in an ideal scenario, are not replicated in practice.

Another key finding was that PAG representatives consistently demonstrated a higher tendency to tolerate side effects, compared to HCPs who, in an ideal scenario, emphasized their priority to minimize patient experience of side effects. For example, 94% of HCPs compared to 71% of PAG representatives consented that patient dissatisfaction with side effects prompt time to switch therapy. Similarly, compared to 75% of HCPs, only 57% of PAG representatives supported the utility for novel treatment for people living with well-controlled gMG but facing minor long-term side effects. Qualitative data suggests that HCPs and PAG representatives differ in their understanding of side effects. While HCPs appeared to interpret side effects as “adverse reactions”, PAG representatives appeared to view it as a “tolerability” issue. Consequently, HCPs strongly supported minimization of side effects, while PAG representative comments suggested that patients may view side effects as a necessary trade-off for symptom control. This difference in interpretation may partially explain the misalignment in opinion consensus, even in an ideal scenario and highlights the need for effective patient-physician communication. Contrarily, these findings may suggest the extent to which gMG symptoms affect QoL, prompting patient willingness to compromise on minor side effects in exchange for better symptom management. Regardless, these findings are supported by prior patient sentiment analysis and surveys which indicate patients often deal with trade-offs in their therapeutic course, indicating their continued reliance on a treatment despite minor side effects [20, 21, 23]. The complex relationship between side effect tolerance and QoL is a particularly nuanced finding, a further consideration for physicians to carefully address during their discussions with patients.

Lastly, an emerging theme in this Delphi project was the importance of patient education. Both HCPs and PAG representatives commented that patients were often too overwhelmed at treatment initiation to actively partake in decision making. However, they also reported that with time, proper education, open discussions, and their own experience of undergoing different treatments, patients can become empowered to actively voice their preferences and champion it. This strengthens the argument for continuity of patient care, which may allow physician-patient relationships to mature over time. Such evolution of physician-patient relationships, in turn, opens avenues for shared and effective decision making.

Importantly, approximately 11% of statements achieved no consensus and some statements achieved only moderate consensus. This varying level of consensus achieved may be influenced by many factors. For example, HCPs and PAGs prior or current experiences along with the patient’s disease course, including their mental health status or disease severity, may influence the time to switch to a new treatment or definition of treatment effectiveness. The moderate consensus achieved for related statements highlights the highly individualized treatment approach that is often used and required in gMG. In addition, participants were asked to respond from an “ideal scenario”, which might vary between individuals and may also limit applicability to real-world clinical settings. Finally, while regulatory approval tends to be uniform across CENE countries, there may be geographical differences in accessibility and cost of therapeutic options, particularly novel therapies, that should be considered when evaluating consensus among HCPs and among PAGs. These differences in accessibility of novel therapeutic options may influence HCPs and PAGs thresholds for treatment switch, and therefore, the consensus achieved within the groups.

In managing chronic and burdensome conditions like gMG, integration of patient needs and preferences is an essential clinical priority. Holistic care of an individual includes not only the identification of an appropriate treatment-dose combination, but also an ongoing dialogue towards mutual understanding, shared goals and approach. This Delphi consensus project found that while HCPs and PAG representatives often align in ideal scenarios, a real-world application of these results is hindered by differences in perception and systemic barriers. This calls for further research and idea generation on ways to practice shared decision-making, particularly in resource-constrained settings to prevent suboptimal outcomes.

## Conclusion

In this Delphi survey, 89% (47 of 53) statements achieved consensus. The results underlined the importance of patient perspectives and QoL as key considerations in gMG management. Consensus outcomes suggest that ideally, HCP perspectives and their understanding of patient perspectives generally align with those of PAG representatives. Nonetheless, contrasting pictures from the real-world highlighted the need for physicians to prioritize regular, structured conversations on QoL, treatment expectations, and side effect profile, alongside symptom control. Qualitative data from comments find that HCPs acknowledge current challenges and express willingness to address them. The results also demonstrate that well-informed patients are more likely to have strong preferences that are openly communicated to physicians. Thus, the findings underscore the need for patients to actively seek information, openly communicate and confidently partake in shared decision making in their own treatment journey. This panel views these outcomes as a meaningful foundation for relevant conversations among the medical, patient and caregiving communities, paving the way for more patient-centric management and care in gMG that goes beyond clinical management and instead reflects a person’s lived experience and priorities. 

## Supplementary Information

Below is the link to the electronic supplementary material.

Supplementary Material 1

## Data Availability

The datasets used and/or analysed during the current study are available from the corresponding author on reasonable request.

## Declarations

### Medical writing and editorial assistance

Medical writing support was provided by our colleagues at Cytel.

## Abbreviations

AChEI: Acetylcholinesterase Inhibitors
AdBoard: Advisory Board
C5i: Complement5 Inhibitors
CENE: Central and Northern Europe
FcRni: Neonatal Fc Receptor Inhibitors
GC: Glucocorticoids
gMG: Generalized Myasthenia Gravis
HCP: Health Care Provider
IgG: Immunoglobulin
IVIg: Intravenous Immunoglobulin
MG: Myasthenia Gravis
NMJ: Neuro Muscular Junction
PAG: Patient Advocacy Group
PLEX: Plasma Replacement/Exchange
QoL: Quality of Life
