# Supplementary material for: Bridging the gap between patient and physician perspectives on management of generalized myasthenia gravis: a Delphi consensus study
Source: Orphanet J Rare Dis. 2026 Mar 20;21:169. doi: 10.1186/s13023-026-04312-7 (PMC13126803; doi:10.1186/s13023-026-04312-7)
Supplement: Supplementary file 1 — Supplementary Material 1 [file 13023_2026_4312_MOESM1_ESM.docx]

| **Study Title:** | Bridging the Gap Between Patient and Physician Perspectives on Management of Generalized Myasthenia Gravis: A Delphi Consensus Study |
| --- | --- |
| **Keywords** | Generalized Myasthenia Gravis, Delphi consensus, quality of life, Europe |
| **Key journal guidelines:** | https://ojrd.biomedcentral.com/submission-guidelines/preparing-your-manuscript/research |
| **Authors** | Andrew Chan*, Monika Kaempf*, Wolfgang N. Loescher, John Vissing, Johan Voerman, Eva Frostell-Pyhäjärvi, Sari Atula |

Table 1: Delphi survey questionnaire and the overall consensus achieved among a total panel of 23 experts

| **N** | **Delphi Survey Question/Statement** | **Consensus (%)** |
| --- | --- | --- |
| 1 | At the time of treatment initiation, patients and physicians openly discuss and align on key aspects of the treatment journey including |  |
|  | a) overall treatment goal for the patient | 91% |
|  | b) treatment goal for the currently prescribed therapy | 91% |
|  | c) waiting time to observe treatment effects | 96% |
|  | d) definition of treatment effectiveness used in assessing treatment outcomes | 74% |
|  | e) time to switch to a new treatment when set goals are not achieved | 83% |
|  | f) potential side effects of currently prescribed therapy | 91% |
| 2 | At the time of treatment initiation, patients are provided with a thorough explanation of first-line treatments |  |
|  | a) all potential treatment options currently available in the market | 78% |
|  | b) their efficacy profiles | 74% |
|  | c) their potential side effects in symptom management | 83% |
|  | d) their response timelines | 87% |
| 3 | At the time of treatment modification, patients are provided with a thorough explanation of |  |
|  | a) all potential treatment options currently available in the market | 74% |
|  | b) their efficacy profiles | 87% |
|  | c) their potential side effects in symptom management | 87% |
|  | d) their response timelines | 91% |
| 4 | Patients and physicians have a shared understanding regarding definition of severe symptoms which they mutually discuss and apply during the treatment process. | 91% |
| 5 | Physicians provide patients with a clear timeline for follow-up assessments during the initial consultation | 83% |
| 6 | Transparent discussions between patients and physicians considerably improve |  |
|  | a) physician understanding of patient preferences and treatment priorities | 83% |
|  | b) patient education about current treatment options and practices | 87% |
| 7 | (Following from above statements) There is a need to enhance the level of open and transparent communication between physicians and patients. | 78% |
| 8 | Patients regard the following highly when selecting a treatment option |  |
|  | a) mode of treatment, whether oral, subcutaneous injection or infusion. | 78% |
|  | b) frequency of treatment administration (e.g., daily, weekly, or monthly) | 78% |
|  | c) potential side effects | 100% |
|  | d) necessity of treatment side effects monitoring (e.g., regular blood tests) | 74% |
|  | e) efficacy of treatment in controlling symptoms | 100% |
| 9 | When selecting gMG treatments, these patient preferences are considered: |  |
|  | a) Dosing frequency | 83% |
|  | b) Route of administration | 91% |
|  | c) Necessity of treatment side effects monitoring (e.g., regular blood tests) | 74% |
|  | d) Sustained symptom control | 100% |
|  | e) Safety | 96% |
| 10 | All patient co-morbidities have an equal impact on treatment decisions | No consensus |
| 11 | Mental health-comorbidities such as depression or anxiety disorder, are equally important as physical health-comorbidities such as diabetes when making treatment decisions | 83% |
| 12 | The treatment goal set for gMG therapy takes into consideration patient burden and needs alongside symptom management. | 78% |
| 13 | The common definition of symptom severity includes the impact on quality of life, rather than focusing solely on clinical manifestations | 74% |
| 14 | In gMG care, the potential for quality-of-life improvement is a compelling reason to initiate or modify treatments, even for patients with mild or stable clinical profiles | 83% |
| 15 | There is utility for novel therapies if gMG symptoms are well controlled and |  |
|  | a) There is limited potential to improve quality of life. | No consensus |
|  | b) There is some potential to improve quality of life. | 74% |
|  | c) There are minor short-term side effects of the current treatment. | No consensus |
|  | d) There are minor long-term side effects of the current treatment. | 70% |
|  | e) There are potential short or long-term side effects of the current treatment. | 83% |
| 16 | When determining the potential for gMG treatment initiation or modification to improve quality of life, following aspects are important components |  |
|  | a) Professional functioning and career impact | 91% |
|  | b) Social well-being and relationships | 91% |
|  | c) Mental health and emotional state | 87% |
|  | d) Physical capabilities and daily functioning | 100% |
|  | e) Overall life satisfaction | 83% |
| 17 | The time between initial gMG diagnosis and achieving an optimal treatment plan is considerably reduced when |  |
|  | a) There are clearly established treatment goals at the time of therapy initiation. | 70% |
|  | b) There are explicitly set timelines for reviewing treatment effectiveness in achieving set treatment goals. | 87% |
| 18 | The following strongly indicates the timing for reevaluating and modifying the current treatment regimen or dosage |  |
|  | a) Unmet treatment goals within set timelines | 78% |
|  | b) Patient dissatisfaction with their quality-of-life | 78% |
|  | c) Patient dissatisfaction with treatment side effects | 87% |
| 19 | Patient dissatisfaction alone is sufficient to consider treatment switch. | No consensus |
| 20 | Patient dissatisfaction with treatment side effects is as important as dissatisfaction with efficacy in guiding treatment modifications. | 74% |
| 21 | For patients with stable disease activity, treatment reevaluation typically occurs every |  |
|  | a) 6-12 months | 96% |
|  | b) >12 months | No consensus |
